# Experimental Evaluation of the Transport Mechanisms of PoIFN-α in Caco-2 Cells

**DOI:** 10.3389/fphar.2017.00781

**Published:** 2017-11-07

**Authors:** Xin Liu, Sidi Zheng, Yue Qin, Wenya Ding, Yabin Tu, Xingru Chen, Yunzhou Wu, Li Yanhua, Xuehui Cai

**Affiliations:** ^1^College of Veterinary Medicine, Northeast Agricultural University, Harbin, China; ^2^Heilongjiang Key Laboratory for Animal Disease Control and Pharmaceutical Development, Harbin, China; ^3^Harbin Veterinary Institute of Chinese Academy of Sciences, Harbin, China

**Keywords:** PoIFN-α, Caco-2 cells, endocytosis, intracellular trafficking, exocytosis, transcytosis

## Abstract

For the development of an efficient intestinal delivery system for Porcine interferon-α (PoIFN-α), the understanding of transport mechanisms of which in the intestinal cell is essential. In this study, we investigated the absorption mechanisms of PoIFN-α in intestine cells. Caco-2 cells and fluorescein isothiocyanate-labeled (FITC)-PoIFN-α were used to explore the whole transport process, including endocytosis, intracellular trafficking, exocytosis, and transcytosis. Via various techniques, the transport pathways of PoIFN-α in Caco-2 cells and the mechanisms were clarified. Firstly, the endocytosis of PoIFN-α by Caco-2 cells was time, concentration and temperature dependence. And the lipid raft/caveolae endocytosis was the most likely endocytic pathway for PoIFN-α. Secondly, both Golgi apparatus and lysosome were involved in the intracellular trafficking of PoIFN-α. Thirdly, the treatment of indomethacin resulted in a significant decrease of exocytosis of PoIFN-α, indicating the participation of cyclooxygenase. Finally, to evaluate the efficiency of PoIFN-α transport, the transepithelial electrical resistance (TEER) value was measured to investigate the tight junctional integrity of the cell monolayers. The fluorescence microscope results revealed that the transport of PoIFN-α across the Caco-2 cell monolayers was restricted. In conclusion, this study depicts a probable picture of PoIFN-α transport in Caco-2 cells characterized by non-specificity, partial energy-dependency and low transcytosis.

## Introduction

Viral diseases lead to severe economical losses in the swine industry. In the clinic, the preventative vaccines have played an important role in many viral diseases under control. However, they are still incomplete for eliminating these virus diseases (Huang et al., 2012). Therefore, efficient antiviral drugs are needed to cure the infected animals. It is known that interferon (IFN)plays a significant role in the host antiviral defense by stimulating the expression of antiviral proteins in the infected host cells (Pfeffer et al., 1998). Porcine interferon-α (PoIFN-α) as one of the type I IFN family members has been widely used to inhibit viral infections, such as Porcine Reproductive and Respiratory Syndrome Virus (PRRSV) (Sang et al., 2010), Vesicular Stomatitis Virus (VSV) (Horisberger, 1992), and Transmissible Gastroenteritis Virus (TGEV) (Jordan and Derbyshire, 1995).

Traditionally, PoIFN-α has been administrated by injection, yet several side effects, such as bad compliance and stress still cause problems in clinical use (Li et al., 2016). On the contrary, oral administration is generally preferred by the veterinarians due to good compliance and superior safety (Yun et al., 2013). Moreover, it is likely that the small intestine may play an important position for the oral administration due to its large surface. In general, there are four distinct mechanisms for drugs to cross the intestinal cell: paracellular, transcellular, carrier-mediated, and receptor-mediated transport. The absorption patterns depend on physical characteristics of drugs, such as molecular weight, hydrophobicity, ionization constants, and pH stability (Yun et al., 2013). The structural features of intestine would be advantageous to absorb small and lipophilic drugs through passive diffusion, but not too large molecular sizes, such as protein or peptide. Therefore, PoIFN-α as a macromolecule protein shows poor oral bioavailability due to its instability in gastrointestinal tract, low permeability across the small intestine (Donovan et al., 1990; Mahato et al., 2003; Shaji and Patole, 2008), as well as short plasma half-life (Saffran et al., 1986; Fix, 1996).

During the last few decades, various strategies have been developed, attempting to enhance the bioavailability of protein drugs, including the use of permeation enhancers (Aungst, 2012), enzyme inhibitors (Yamamoto et al., 1994), and different drug delivery systems (Sarmento et al., 2007; Sharma et al., 2010; Niu et al., 2011). However, the mechanisms and underlying pathways of proteins, especially PoIFN-α, transport through the intestinal epithelial cells have not yet been fully elucidated.

In modern drug delivery researches, cell culture models represent a valuable tool to study the oral delivery of drugs, peptides, or vaccines (Artursson et al., 2012). The vast majority of the model of intestinal absorption has been performed using Caco-2 cells which were originated from human epithelial colorectal adenocarcinoma cells. The model was recommended by both the US FDA and EMA as the most suitable model for estimating intestinal permeability. Recently, Caco-2 cells have been widely used to evaluate the efficiency and elucidate cellular mechanisms behind the adsorption of micelles (Abramov et al., 2015) and polymeric particles (Alai and Lin, 2015; Gossmann et al., 2015).

In this study, in order to understand the transport of PoIFN-α in the intestine, we used Caco-2 cells as the *in vitro* model to investigate different transport processes including endocytosis, intracellular trafficking, exocytosis and transcytosis, with various quantitative and qualitative techniques.

## Materials and methods

### Materials

Recombinant porcine IFN-α was obtained as a gift sample from Harbin Veterinary Institute of Chinese Academy of Sciences. Caco-2 cell line (Human colon carcinoma) was purchased from Stem Cell Bank, Chinese Academy of Sciences. High Glucose DMEM and Hank's balanced salt solution (HBSS) were obtained from Gibco, USA. HOOK^TM^-Dye Labeling Kit was purchased from Biosciences, USA. ALP Kit was purchased from Jiancheng Bioengineering institute, Nanjing, China. Trypsin-EDTA, penicillin and streptomycin solution, glutamine and non-essential amino acids all were obtained from Hyclone, USA. Fetal bovine serum (FBS) was a product of PAN Biotech, Germany. 12-well Transwell inserts (0.4 μm pore size, 1.13 cm^2^ surface area), 6-, 12-, and 96-well plates all were purchased from Costar, Corning, USA. MTT assay dye and DMSO were purchased from Amresco, USA. Hoechst 33,258 was purchased from Solarbio, China. Chlorpromazine, β-cyclodextrin, Wortmannin and Amiloride were all purchased from Sigma-Aldrich, USA.

### Preparation of FITC-labelled PoIFN-α

In order to readily detection, fluorescein isothiocyanate-labeled (FITC) was labeled in PoIFN-α. The procedure of labeling PoIFN-α was prepared according to the manufacture's instructions of the HOOK^TM^ Dye Labeling Kit. Briefly, the freshly prepared Dye Labeling Agent solution was added to the PoIFN-α solution. Quickly, the mixture was treated violently and then incubated at room temperature under dark condition for 60 min. Then, the prepared mixture was transferred to the SpinOut^TM^ GT-600 column, which was used to remove the unconjugated dye. The column containing mixture was placed in 15 mL centrifuge collection tube and centrifuged at 1,000 × g for 4 min. Finally, the purified FITC- PoIFN-α in the tube was frozen at −80°C and protected from light for the following assays.

### Cell culture model

Caco-2 cells were maintained in T-25-cm^2^ flasks at 37°C in an atmosphere of 5% CO_2_/5% air and 90% relative humidity. DMEM medium (high glucose, GIBCO) supplemented with 20%fetal serum bovine, 1% non-essential amino acids, 1% L-glutamine, 1% penicillin-streptomycin solution was used as culture medium. Cells were trypsinized when reaching 80–90% confluency and were seeded in 6- and 96-well plate respectively.

### Viability assay

MTT assay was utilized here to evaluate the influence of FITC- PoIFN-α on cell viability. Caco-2 cells seeded in 96-well plate at density of 1.5 × 10^5^/mL were cultured for 24 h. The viability assay was carried out as described previously with minor modifications (Joshi et al., 2016). Briefly, FITC-PoIFN-α was diluted with complete medium with different concentration (5, 10, 20, 40, 80, and 100 μg/mL). Then, 200 μL samples were added into each well with eight times repeats for each concentration, and the medium was used as negative control. After 6 h of incubation, 150 μL of MTT [3-(4,5)-dimethylthiahiazo (-z-y1)-3,5-di- phenytetrazoliumromide] reagent (1 mg/mL) in PBS medium was added to each well. Then 96-well plates were incubated at 37°C for 4 h. After the incubation period, the supernatant was discarded, and 150 μL DMSO was added into each well to dissolve the intracellular formazan. Lastly, the results were obtained on a microplate multi-detection instrument by quantifying the absorbance wavelength at 590 nm. The percentage of cell viability was calculated based on the absorbance of treated cells against the medium treated negative control.

### Uptake characterization of FITC-PoIFN-α

#### Qualitative assays

Caco-2 cells at the density of 1.7 × 10^5^/mL were seeded in 6-well plates and glass-Bottom Dishes respectively for 14 days. Before uptake experiments, the medium was replaced with 37°C HBSS and incubated at 37°C for 30 min. Then, the preheating HBSS was used to wash Caco-2 cells for 3 times, and then 20 μg/mL FITC-PoIFN-α was put into Caco-2 cells for 6 h. Following rinsed in cold HBSS twice, the cells were fixed by 4% paraformaldehyde at room temperature for 15 min and stained by 10 μg/mL Hochest 33,258 at room temperature for 5 min to mark cell nucleus. Results were obtained using Fluorescence Microscope and High Resolution Microscope (DeltaVision OMX Blaze™, GE, USA) under 488 and 346 nm excitation.

#### Quantitative assays

The quantitative characterization of Caco-2 cells uptake was measured by Flow Cytometry System (FCS) (BD FACSAriaTM IIu Cell Sorter, BD Biosciences, USA) (He et al., 2013). Caco-2 cells at density of 1.5 × 10^5^/mL were seeded in 6-well plates and cultured for 14 days. Before the quantitative assays, Caco-2 cells were washed by 37°C HBSS for 3 times and were incubated with medium containing FITC-PoIFN-α at 5, 10, and 20 μg/mL. At the 2, 4, and 6 h, Caco-2 cells were digested, washed with cold HBSS for 3 times. For each sample, 10^6^ cells were sorted by FCS. Additionally, Caco-2 cells treated with only medium were used as negative controls.

#### Temperature-dependent of FITC-PoIFN-α uptake

To investigate the temperature-dependent of FITC-PoIFN-α uptake, the FCS was used to quantitate the mean intracellular fluorescence intensity of cells (He et al., 2013). The assay was performed as described previously (Ikehata et al., 2009). Briefly, Caco-2 cells were cultured in 6-well plate for 14 days. Then, the cells were washed with cold HBSS for 3 times, and 20 μg/mL FITC-PoIFN-α was added to each well, following 2 h incubation time at 37 and 4°C, respectively. Finally, the cells on the plates were trypsinized, washed by cold HBSS twice and resuspended in 500 μL PBS. For each sample, 10^6^ cells were sorted by FCS. Cells culture with only medium was served as the negative control.

### Endocytosis pathway of FITC- PoIFN-α in Caco-2 cells

The endocytosis pathways of FITC- PoIFN-α were investigated with the specific pharmacological inhibitors as described in Table 1. Caco-2 cells grown in 6-well plate were pre-incubated with the inhibitors for 30 min. Then 20 μg/mL FITC-PoIFN-α was added to the culture. After 4 h, Caco-2 cells uptake was terminated by cold HBSS. Then the cells on the plates were trypsinized, washed by cold HBSS twice and resuspended in 500 μL PBS. For each sample, 10^6^ cells were sorted by FCS.

**Table 1:** Inhibitors used in the pathway assay with different functions and their concentrations.

**Inhibitors**	**Concentrations**	**Functions**
Chlorpromazine	25 μM	Inhibition of clathrin-mediated endocytsis by accelerating decomposition of clathrin.
β-cyclodextrin	10 mM	Cholesterol extraction agent. Effective inhibition of lipid/raft pathway. Inhibitor of clathrin-mediated pathway.
wortmannin	200 nM	Inhibition of Macropinocytosis pathway by suppressing formation of macropinocytosis bodies. Inhibitor of clathrin-mediated endocytsis.
Amiloride	2.5 mM	Inhibition of Macropinocytosis pathway by specifically blocking the Na+/H+ exchanger.
Brefeldin A	25 mg/mL	The Golgi apparatus/ER related inhibitor.
Monensin	33 mg/mL	The Golgi apparatus related inhibitor.
LY294002	1 mM	The Lysosomal related inhibitor.
Nocodazole	10 mM	The microtubule related inhibitor.
Indomethacin	300 mM	The COX related inhibitor.

### Intracellular trafficking of FITC-PoIFN-α in Caco-2 cells

For the location detection of intracellular FITC-PoIFN-α, we reasonably used unspecific inhibitors (Table 1). Caco-2 cells were incubated with 20 μg/mL FITC-PoIFN-α for 2 h, and then washed with cold HBSS 3 times after suspensions removed. Subsequently, the cells were incubated with medium respectively containing brefeldin A (25 mg/ml), monensin (32.5 mg/ml) and LY294002 (1 mM) for another 4 h. At last, the cells on the plates were trypsinized, washed by cold HBSS twice and resuspended in 500 μL PBS. The amount of FITC- PoIFN-α in Caco-2 cells was measured as described previously (Xu et al., 2016).

### Exocytosis detection of FITC-PoIFN-α from Caco-2 cells

In the exocytosis mechanism investigation, the specific inhibitors were shown in Table 1. Caco-2 cells were firstly incubated with 20 μg/mL FITC-PoIFN-α for 2 h. Then, caco-2 cells were washed by cold HBSS three time, and only medium, Indomethacin(300 mM), and Nocodazole(10 μM) was added for subsequent re-incubation. After 4 h incubation, the cells on the plates were trypsinized, washed by cold HBSS twice and resuspended in 500 μL PBS. The intracellular FITC-PoIFN-α were finally detected by the FCS as the same method described above (Xu et al., 2016).

### Transportation of FITC-PoIFN-α across Caco-2 cells monolayer

To measure the transportation of FITC-PoIFN-α to basal side, Caco-2 cells were plated in transwell as reported earlier (Li et al., 2016). Briefly, Caco-2 cells at the density of 3.0 × 10^5^/mL were seeded in 12-well transwell membrane for 21 days. The medium was changed every other day for the first week, and then it was replaced every day until the 21 days (Zhou et al., 2005). Before permeability studies, transepithelial electrical resistance (TEER) (Wang et al., 2008) (Millicell-ERS, Millipore, USA) and the activity of alkaline phosphatase (Ng et al., 2004; Ghaffarian and Muro, 2013) were detected. Before the transport assays, Caco-2 cell monolayers plated in transwell membrane were washed with 37°C HBSS 3 times. Then, 20 μg/mL of FITC-PoIFN-α was added into the apical side, and 1.5 mL HBSS to the basolateral side. 100 μL samples were taken out from the basolateral side at 30, 60, and 90 min respectively, which were replaced by the same volume of 37°C HBSS. Before and after transport assay, TEER was tested to guarantee the intact of monolayer. Besides the experiment groups, the FITC- PoIFN-α crossed the transwell insert without Caco-2 cell monolayer was served as positive control, whereas HBSS crossed the Caco-2 cell monolayers was used for the blank control. Samples with FITC-PoIFN-α were measured by Fluorescence Microscope. All the assays were repeated 3 times.

## Data analysis

Values were expressed as means ± SDs. The statistical difference among different groups were compared by 1-way ANOVA followed by a Tukey and *p* < 0.05 considered statistically significant.

## Results and discussion

### Effect of FITC-PoIFN-α on Caco-2 cells viability

It has been shown that PoIFN-α has the effect of anti-tumorigenesis, and possibly inhibits the growth of Caco-2 cells which originates from colon carcinoma cell. In order to confirm the inhibition of FITC- PoIFN-α, we performed the MTT assay. Upto concentration of 20 μg/mL, the FITC-PoIFN-α did not exhibit inhibition (Supplementary Figure 1), and it offered the dosage to the following assays.

### Uptake assays of FITC-PoIFN-α in Caco-2 cells

**Figure 1:**
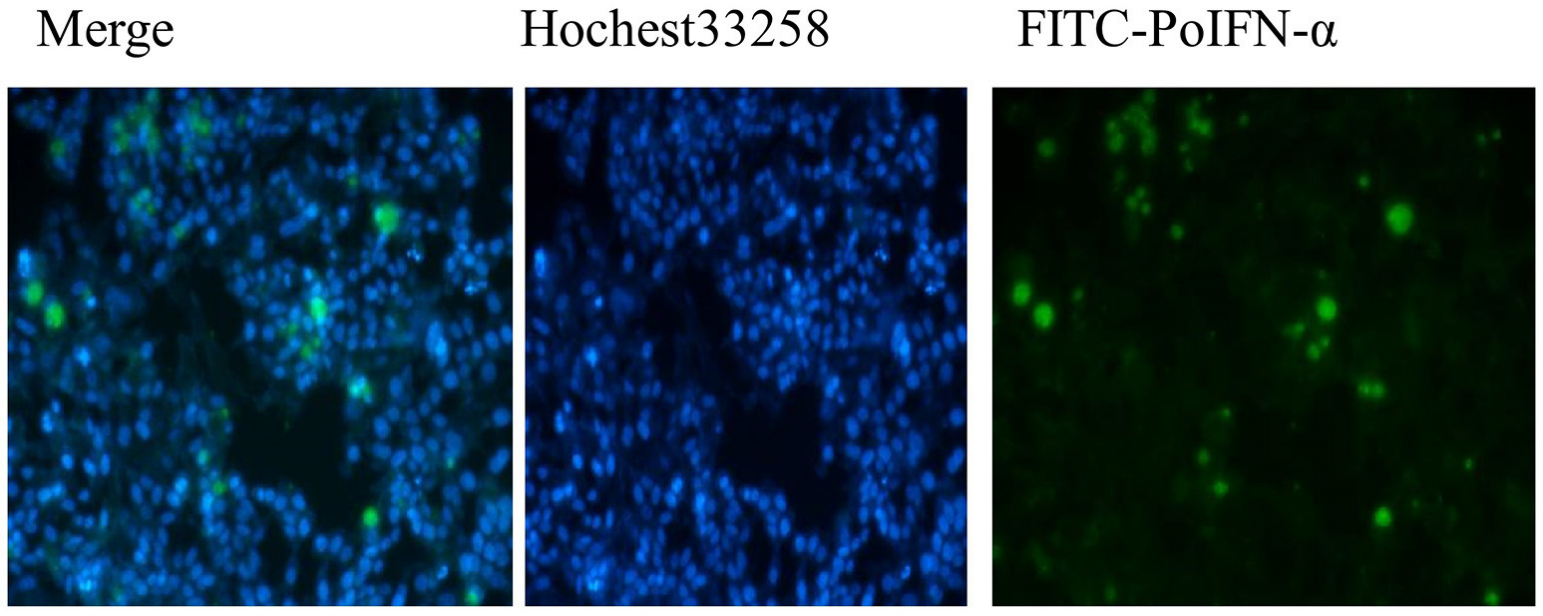
Qualitative Caco-2 cells uptake of FITC-PoIFN-α. Flueorescence micrographs of Caco-2 cells incubated with 20 μg/mL FITC-PoIFN-α for 6 h.

**Figure 2:**
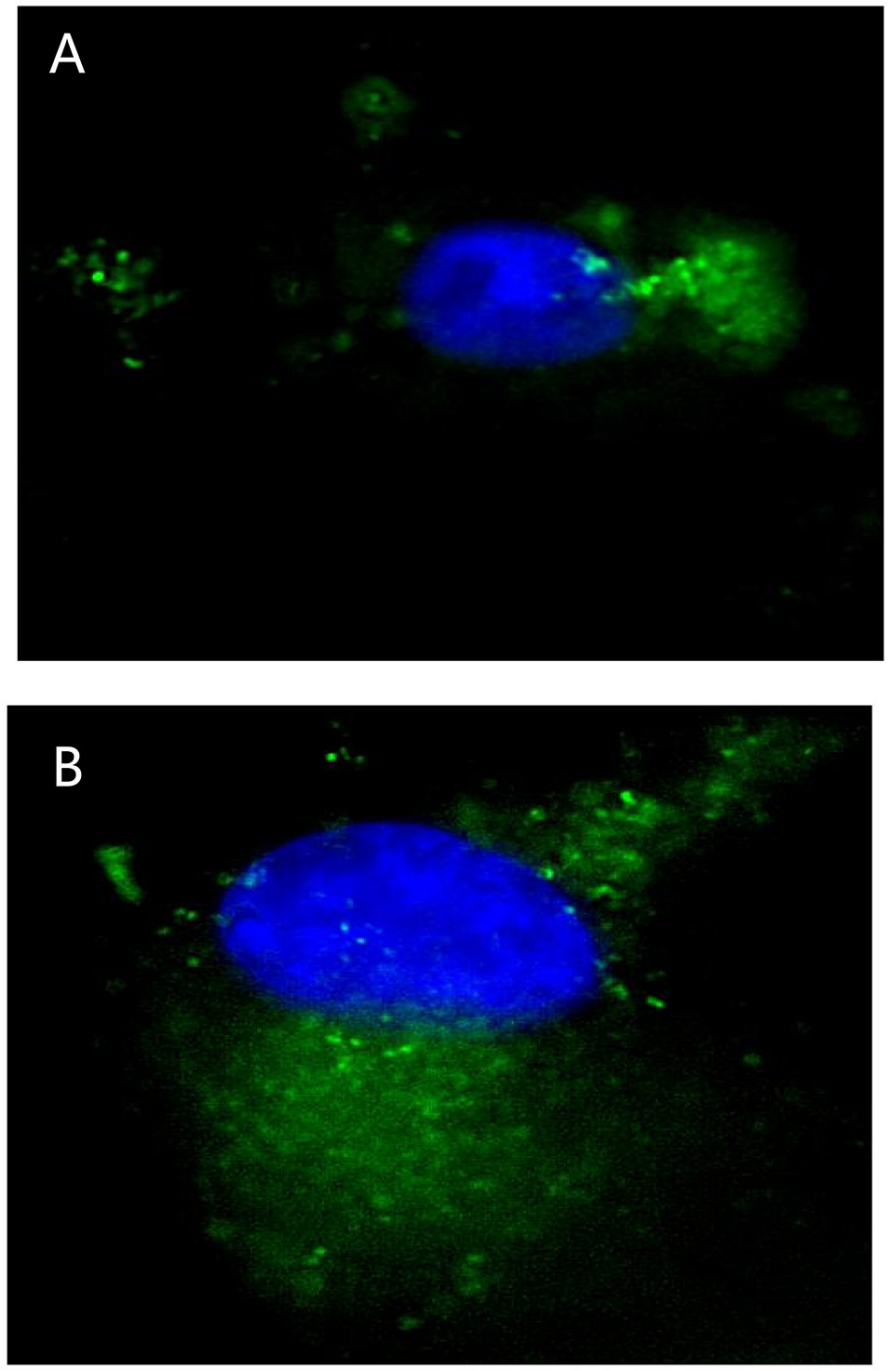
Micrographs obtained by high resolution microscope in Caco-2 cells uptake of 20 μg/mL FITC-PoIFN-α incubated with for 6 h. Panels **(A,B)** stand for different Caco-2 cells uptake the FITC-PoIFN-α. The cell nucleus was dyed in blue, and the FITC-PoIFN-α presented in green.

The Fluorescence Microscope images showed that FITC signals gathered around the nucleus, which demonstrated the FITC- PoIFN-α was most likely absorbed into the Caco-2 cells (Figure 1). Furthermore, in a higher magnification, as shown in Figures 2A,B, the position of FITC signals was extremely close to the nucleus, suggesting that FITC-PoIFN-α could be transported inside of the Caco-2 cells. The previous study has proposed peptides and proteins can be absorbed across the different epithelium by endocytosis pathway (Kim and Malik, 2003). Accordingly, we could clearly observe the uptake of FITC-PoIFN-α by the cells. However, the FITC signals were not strong. It was likely due to the poor permeability for protein drugs (Liu et al., 2016). Moreover, it might also explain the low bioavailability of protein drugs.

### Time-dependent and concentration-dependent uptake of FITC-PoIFN-α in Caco-2 cells

**Figure 3:**
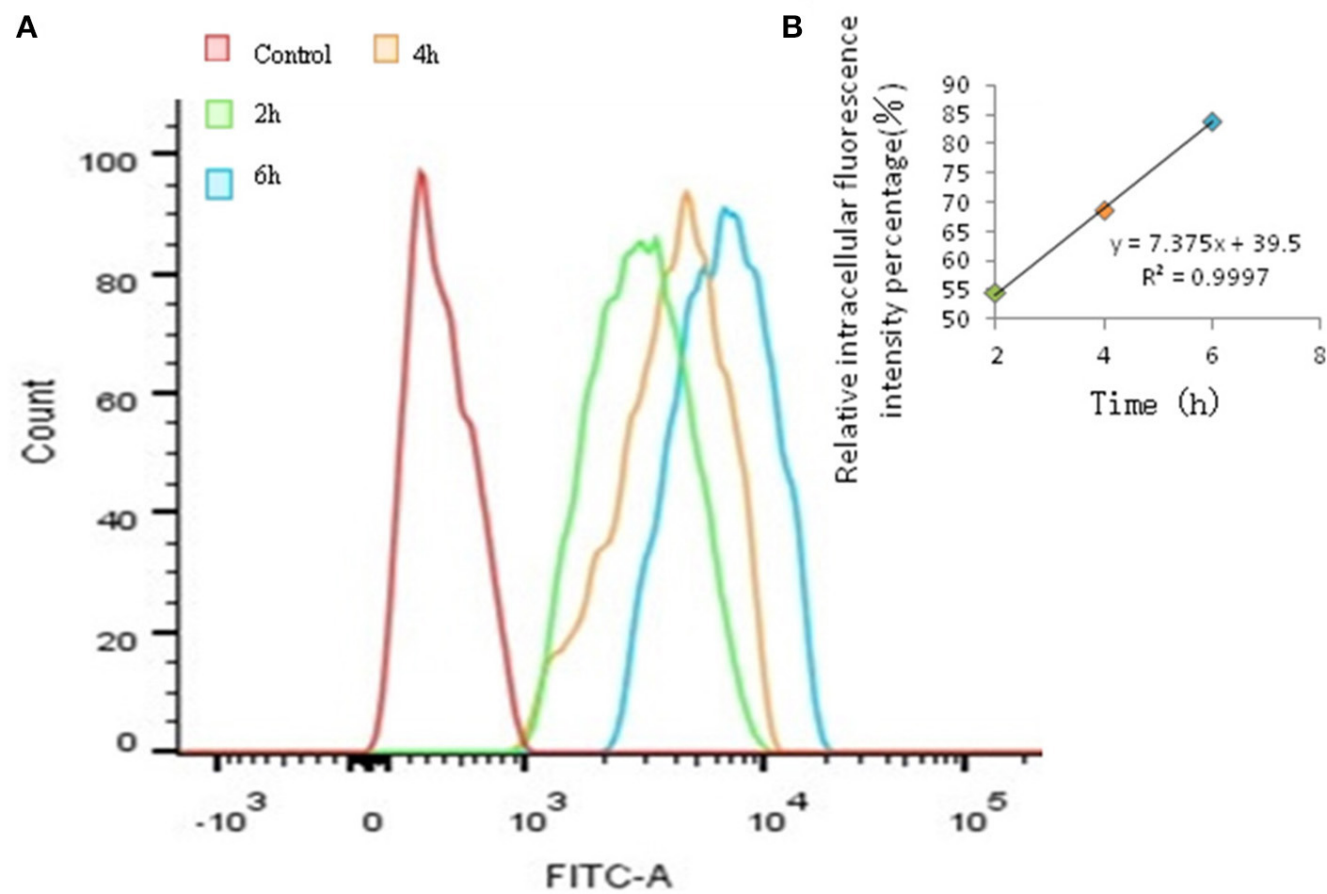
Quantitative Caco-2 cells uptake of FITC-PoIFN-α by FCS. **(A)** Time-dependence of Caco-2 cells uptake of FITC-PoIFN-α. After the incubation with FITC-PoIFN-α (20 μg/mL) for 2, 4, and 6 h, flueorescence in Caco-2 cells measured. **(B)** The liner correlation of time and relative intracellular fluorescence intensity percentage. After the incubation with FITC-PoIFN-α (20 μg/mL) for 2, 4, and 6 h, relative fluorescence intensity in caco-2 cells measured.

The time-dependent and concentration-dependent uptake of FITC- PoIFN-α were investigated. With the extension of incubation in the same concentration of FITC-PoIFN-α, the peak of fluorescence intensity shifted to the right compared to the control (Figure 3A). Namely, the intracellular fluorescence intensity of FITC-PoIFN-α significantly increased. Meanwhile, the correlation of time and relative intracellular fluorescence intensity percentage was investigated in Figure 3B. The results showed the linear correlation with the cultural time going on. So the above figures demonstrated that the uptake of FITC- PoIFN-α was time dependent.

**Figure 4:**
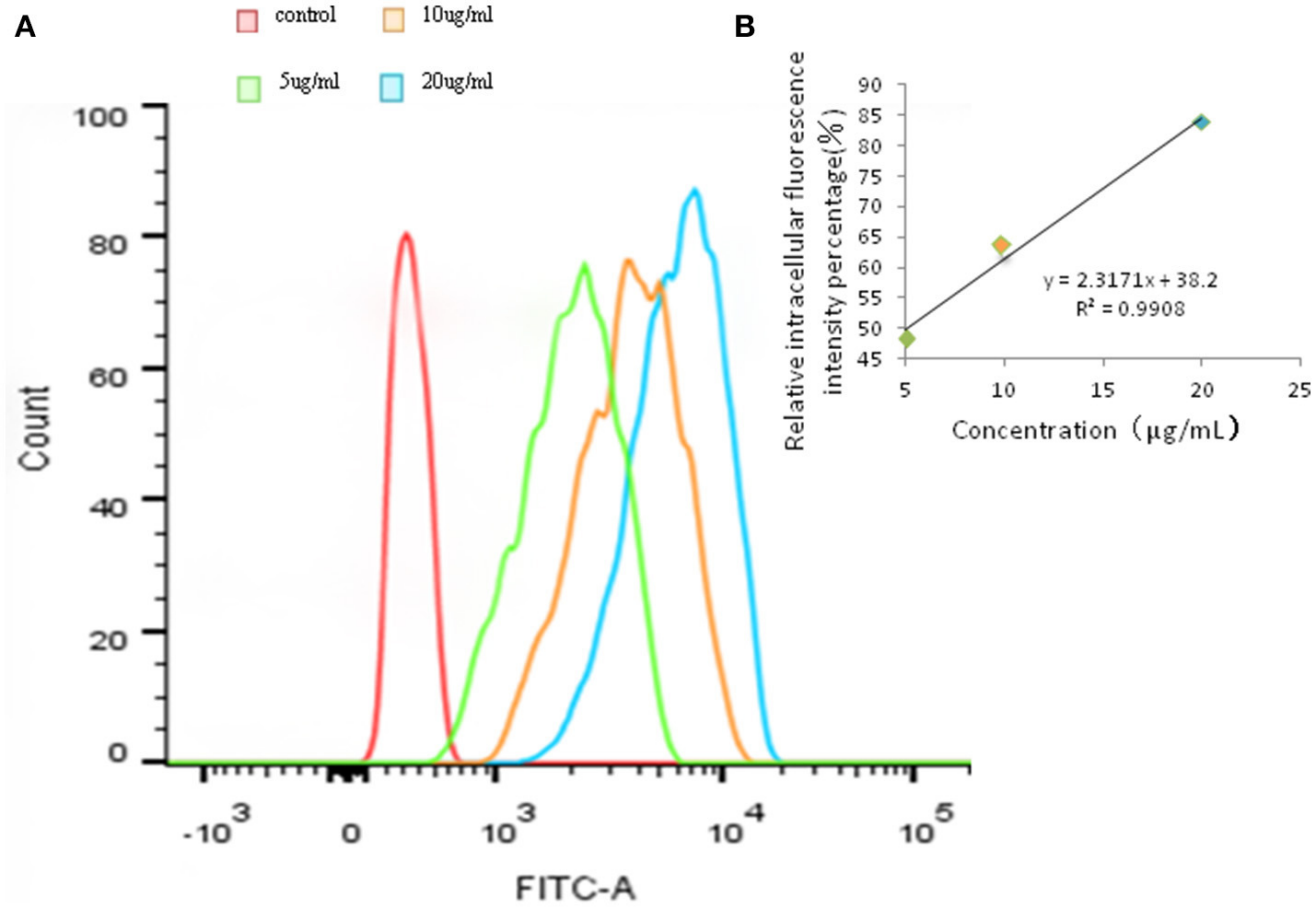
Quantitative Caco-2 cells uptake of FITC-PoIFN-α by FCS. **(A)** Concentration-dependence of Caco-2 cells uptake of FITC-PoIFN-α. After the incubation with different concentration of FITC-PoIFN-α (5,10, and 20 μg/mL) for 6 h, flueorescence in Caco-2 cells measured. **(B)** The liner correlation of concentration and relative intracellular fluorescence intensity percentage. After the incubation with different concentration of FITC-PoIFN-α (5,10, and 20 μg/mL) for 6 h, relative fluorescence intensity in Caco-2 cells measured.

Addtionally, the same trend appeared in Figures 4A,B. Hence, these results suggesting that the uptake of FITC-PoIFN-α in Caco-2 cells is concentration dependent.

### Partially energy-dependent uptake of FITC-PoIFN-α in Caco-2 cells

**Figure 5:**
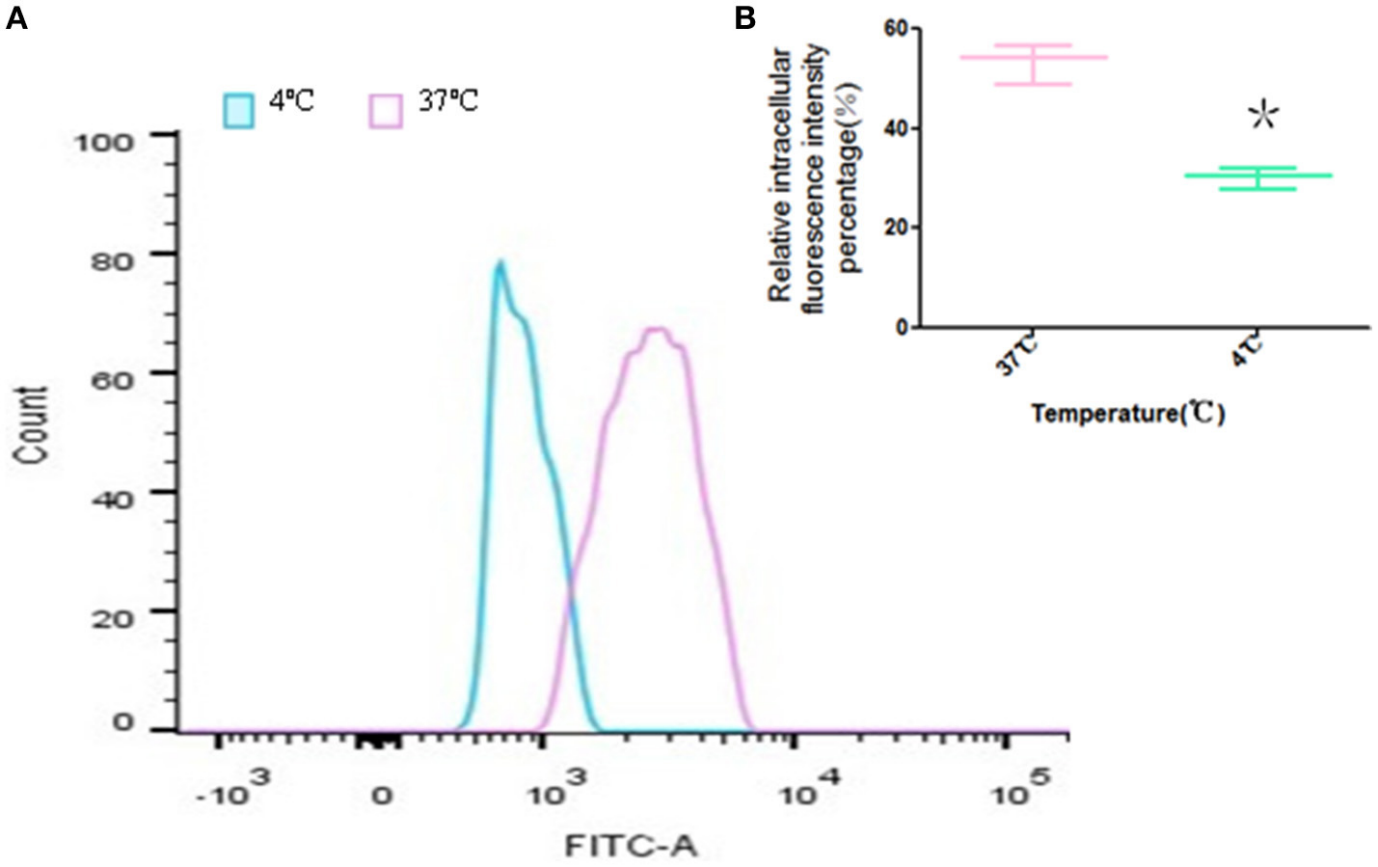
Temperature-dependence of Caco-2 cells uptake of FITC-PoIFN-α. **(A)** Uptake of Caco-2 cells incubated with FITC-PoIFN-α (20 μg/mL) for 2 h at 37 and 4°C. **(B)** The relative intracellular fluorescence intensity percentage measured at 37 and 4°C, respectively (mean S.D., *n* = 3). ^*^p < 0.05 vs. control.

The ability of the cellular uptake of FITC-PoIFN-α in Caco-2 cells at 37 or 4°C was compared via the detection of the intracellular fluorescence. The results showed that the peak of the fluorescence intensity was shifted significantly when the FITC-PoIFN-α treated Caco-2 cells were incubated at 37°C, vs. the cells with the same treatment which were incubated in 4°C (Figure 5A). At the same time, the statistical analysis showed that relative fluorescence intensity percentage of Caco-2 cells at 4°C distinctively decreased (*p* < 0.05; Figure 5B). So, the results also illustrated that the uptake of FITC-PoIFN-α in Caco-2 cells was partially energy-dependent. As a key factor, the temperature significantly influences the ligand-receptor interaction, the internalization of ligand-receptor complexes, and the bioactivities of multiple membrane proteins (Tzafriri et al., 2004). Therefore, temperature-dependency is usually regarded as an index of energy-dependency macromolecules (He et al., 2013).

### Endocytosis pathway of FITC-PoIFN-α

**Figure 6:**
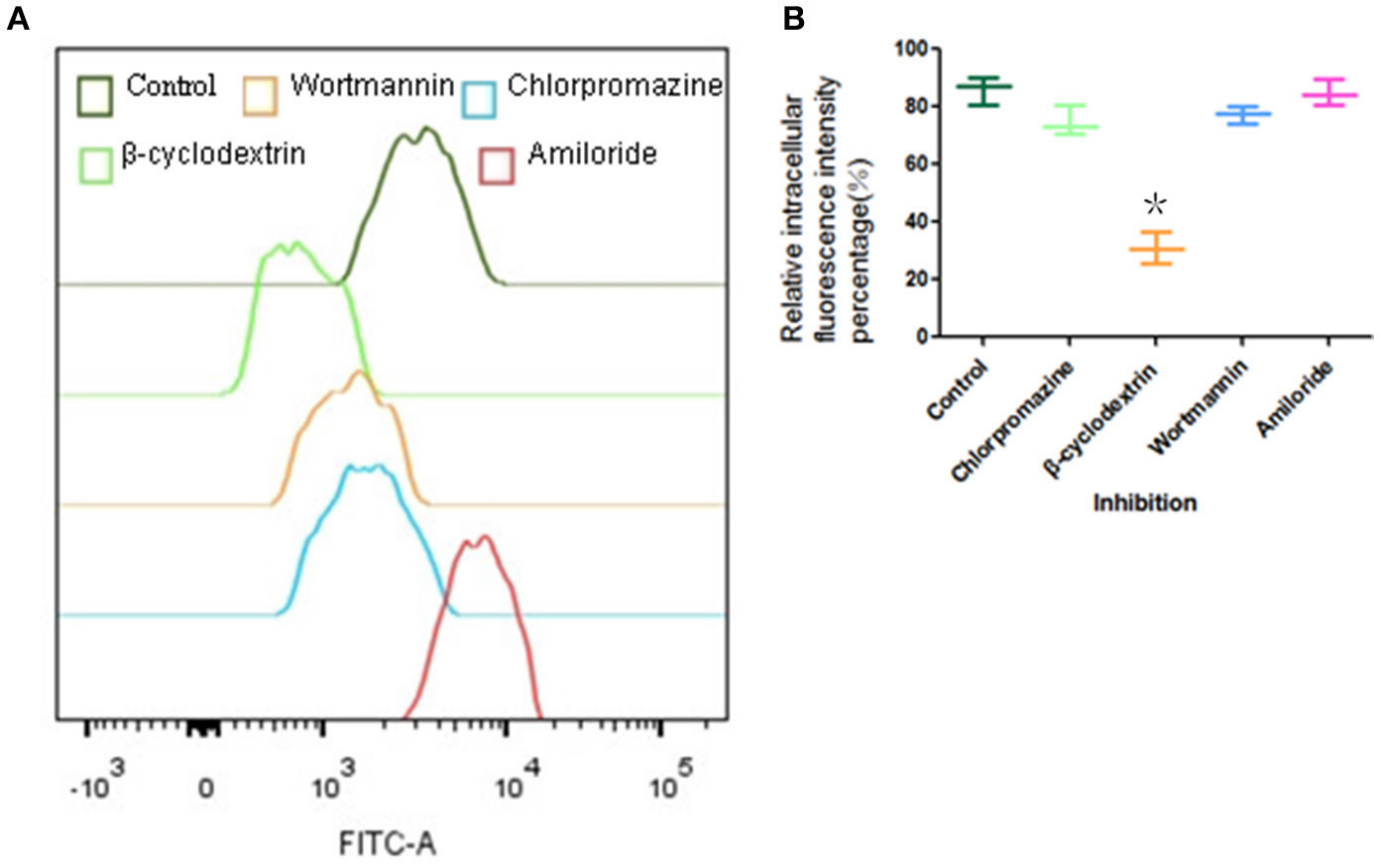
Endocytic pathway of Caco-2 cells uptake of FITC-PoIFN-α. Influence of I ibitors (Chlorpromazine, β-cyclodextrin and wortmannin)in Caco-2 cells uptake of FITC-PoIFN-α. **(A)** Incubated with inhibitors, the retention mean fluorescence intensity of FITC- PoIFN-α in Caco-2 cells. **(B)** The relative intracellular fluorescence intensity percentage in Caco-2 cells measured incubated with inhibitions (mean S.D., *n* = 3). ^*^p < 0.05 vs. control.

The effect of various endocytosis inhibitors on the uptake of FITC-PoIFN-α was examined (Figure 6). β-cyclodextrin, an inhibitor of lipid raft /caveolae endocytosis, obviously inhibited FITC -PoIFN-α uptake. An insignificant inhibitory effect was observed on the treatment with chlorpromazine and wortmannin (Figure 6A). Simultaneously, the statistical analysis showed that relative fluorescence intensity percentage of Caco-2 cells inhibited by β-cyclodextrin significantly decreased (*p*<0.05). Therefore, lipid raft /caveolae endocytosis was the most likely endocytic pathway for FITC-PoIFN-α. Interestingly, an increase in cellular uptake of FITC-PoIFN-α was observed after treatment of amiloride. Similar results of increased endocytosis after treatment with the inhibitor have also been reported previously (Perumal et al., 2008; Du et al., 2013)

There are at least four mechanism for the endocytic pathways: clathrin-mediated endocytosis (CME), lipid/raft caveolae-mediated endocytosis (CvME), macropinocytosis, and other types of endocytosis, such as clathrin- and lipid/raft caveolae- independent endocytosis (Oda et al., 2011). Firstly, the clathrin-mediated endocytosis via specific receptor-ligand interaction forms clathrin-coated vesicles, then the coated pits invaginate and form endocytic vesicles and finally fuse with lysosomes. The lysosomes have a highly acidic environment (pH 4.5–5.5) and contain numerous enzymes that can endanger the integrity of the cargo within the cells (Gamboa and Leong, 2013). Secondly, lipid/raft caveolae-mediated endocytosis (CvME) is the predominant mechanism of endocytosis in most cells (Hillaireau and Couvreur, 2009), which occurs from membrane domains rich in cholesterol and sphingolipids and are lined by caveolin - a dimeric protein (de Garibay et al., 2013). On the other hand, the pathway could avoid the lysosomal degradation. Hence, CvME represents the alternative route to target the cargo which is sensitive to enzymes (e.g., peptides, proteins, nucleic acids). Finally, macropinocytosis is a fluid-phase endocytosis that depends on the solute concentration surrounding the cell (Gamboa and Leong, 2013) and generates relatively large vesicles (1–5 μm). Above all, we investigated the uptake mechanism of FITC-PoIFN-α focusing on three types of endocytosis:clathrin-mediated endocytosis, lipid/raft caveolae-mediated endocytosis, and macropinocytosis. The functions of inhibitors are summarized in Table 1. Chlorpromazine as the inhibitor of CME (Jin et al., 2012; Beloqui et al., 2013) did not reduce the internalization of FITC-PoIFN-α, which indicated that the uptake of FITC-PoIFN-α does not belong to the CME. Moreover, it was clear that the internalization of FITC-PoIFN-α was significantly decreased, based on the inhibition effect of β-cyclodextrin, the inhibitor of lipid/raft caveolae-mediated and CME pathway (Lajoie and Nabi, 2007). Taking together the above results excluded the CME, therefore the internalization of FITC-PoIFN-α was likely mediated by lipid/raft caveolae-mediated pathway. Additionally, wortmannin as the inhibitor of macropinocytosis and CME (Damm et al., 2005; Wipf and Halter, 2005) also did not lessen the cellular uptake of FITC-PoIFN-α, revealing the existence of the two pathways. On the contrary, amiloride as another inhibitor of macropinocytosis (Sarkar et al., 2005; Sasahara et al., 2013) enhances the cellular uptake of FITC-PoIFN-α, demonstrating that other endocytosis sub pathways might be activated and compensated the inhibited pathway. Although, the endocytosis mechanism of FITC-PoIFN-α is complicated and may involve multiple subpathways, In this study, we have characterized the lipid/raft caveolae-mediated pathway might be the most likely endocytic pathway for the uptake of FITC- PoIFN-α.

### Intracellular trafficking of FITC-PoIFN-α in Caco-2 cells

**Figure 7:**
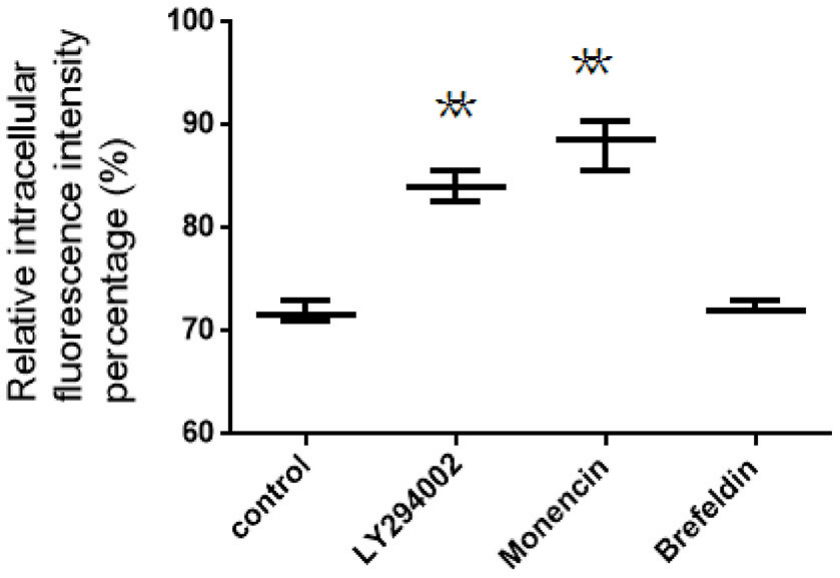
The relative intracellular fluorescence intensity percentage in Caco-2 cells after incubation with unspecific inhibitors (mean S.D., *n* = 3). ^**^p < 0.01 vs. control.

The intracellular location of FITC-PoIFN-α in lysosomes, endoplasmic reticulum (ER) and Golgi apparatus can be assumed by the application of organelle unspecific inhibitors. As shown from the relative intracellular fluorescence intensity percentage (Figure 7), the retention of FITC-PoIFN-α in Coca-2 cells was significantly increased after the incubation with LY294002, suggesting that lysosome acted as an important regulator for the transport of internalized particles (Davidson, 1995).

Besides, the inhibitors, including brefeldin A and monensin, were utilized to investigate the role of Golgi apparatus and endoplasmic reticulum on intracellular transport of both FITC-PoIFN-α. On the one hand, Monensin, as the inhibitor of Golgi apparatus to plasma membrane through blocking macromolecules via disrupting Golgi complex (Kuismanen et al., 1985), could significantly enhance the cellular accumulation of FITC-PoIFN-α (Figure 7). On the other hand, brefeldin A as the inhibitor of the endoplasmic reticulum/ Golgi apparatus pathway through retrograding transport of Golgi enzymes back to endoplasmic reticulum, did not cause the significant changes (Figure 7; Lippincott-Schwartz et al., 1989). The above results demonstrated that the endoplasmic reticulum/Golgi apparatus pathway play a minor role in the transportation of FITC-PoIFN-α.

### Exocytosis detection of FITC-PoIFN-α from Caco-2 cells

**Figure 8:**
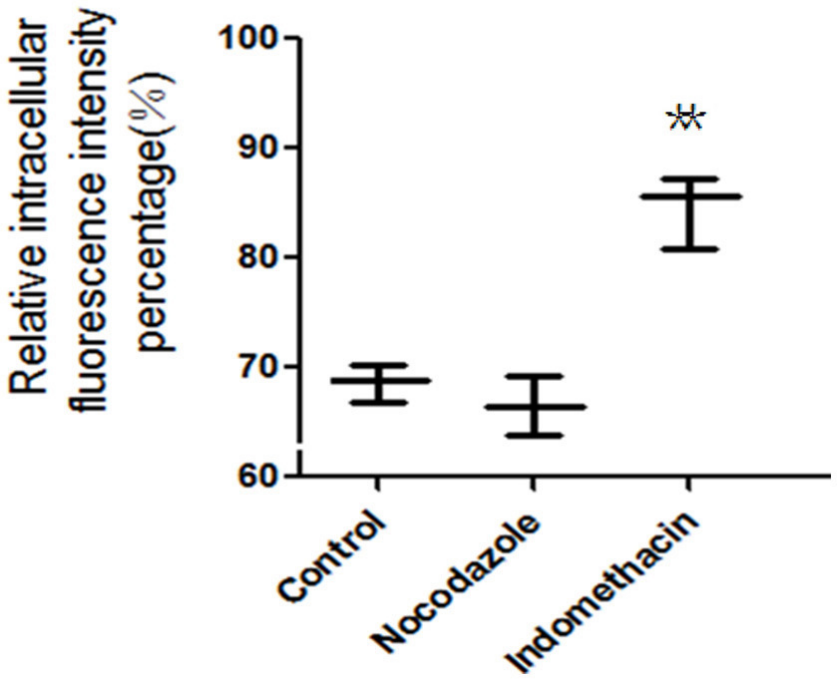
The relative intracellular fluorescence intensity percentage in Caco-2 cells after incubation with unspecific inhibitors (mean S.D., *n* = 3). ^**^p < 0.01 vs. control.

The fusion of intracellular vesicles with the apical or basolateral plasma membrane is defined as exocytosis (Kondor-Koch et al., 1985). In order to simplify the situation, exocytosis here was specifically defined as the upward transportation of endocytosed FITC-PoIFN-α across apical plasma membrane (PM). The amount of FITC- PoIFN-α measured in the Caco-2 cells could indirectly represent that exocytosis of PoIFN-α across Caco-2 cells. FCS was used here to evaluate the effect of microtubule and cyclooxygenase (COX) on exocytosis (Figure 8). Compared with control group, indomethacin could obviously increase the amount of intracellular FITC- PoIFN-α, while Nocodazole did not have any influence.

Indomethacin is a common inhibitor of cyclooxygenase (COX), which catalyzes the oxygenation of arachidonic acid (AA) to form prostaglandin G2 (PGG2) as well as the metabolism of PGG2 to PGH2 (Liou et al., 2001). AA is essential to activate the protein kinase C that involves the exocytosis of some proteins, and the above results indicated that COX might participate in the exocytosis of FITC- PoIFN-α in some degree. Furthermore, the involvement of COX-2 has been confirmed in the exocytosis of certain proteins(Friis et al., 2009). Moreover, to further understand to the exocytosis, nocodazole was also used to specifically blocking the microtubule, which are necessary for the domain-specific fusion in the plasma membrane(Cole and Lippincott-Schwartz, 1995; Schmoranzer et al., 2003). After treatment with Nocodazole, the intracellular FITC- PoIFN-α exhibited no changes, indicating that microtubule uninvolved in exocytosis of FITC- PoIFN-α.

### Determination of Caco-2 cell monolayers model

The values of TEER measured by resistance instrument was elevated with the cultural time, and reached 300 Ω.cm^2^ after cultivating 17 days, which indicated that Caco-2 cells formed a tight monolayer (Supplementary Figure 2). It has been shown that TEER was an effective index for the opening of cells junction or integrity of cell monolayer (Du et al., 2013). Furthermore, the TEER values of monolayers observed in our study were consistent with the reports by others (Wang et al., 2008) However, large variations of TEER values ranging from 200 to 1,000 Ω.cm^2^ have been reported for Caco-2 cells (Hidalgo et al., 1989; Wang et al., 2008). This could possibly be due to Caco-2 cells from different sources variations in growth conditions, which differ greatly in their properties (Hayeshi et al., 2008). The activity of alkaline phosphatase (ALP) results (Supplementary Table 1) showed that the ratio of apical and basal chamber was above 2.5. The manifested differentiation of Caco-2 cells reached to the experimental standard. As one of the markers of well-differentiated Caco-2 cells metaplasia (Ng et al., 2004; Ghaffarian and Muro, 2013), the activity of ALP in apical and basal chamber changed with the cells culture overtime. It indicated that Caco-2 cell monolayer presented the polarized characterization. Hence, the method above can be used to evaluate the possibility of drug delivery for Caco-2 cell monolayers model.

### Transportation of FITC-PoIFN-α across Caco-2 cell monolayers

**Figure 9:**
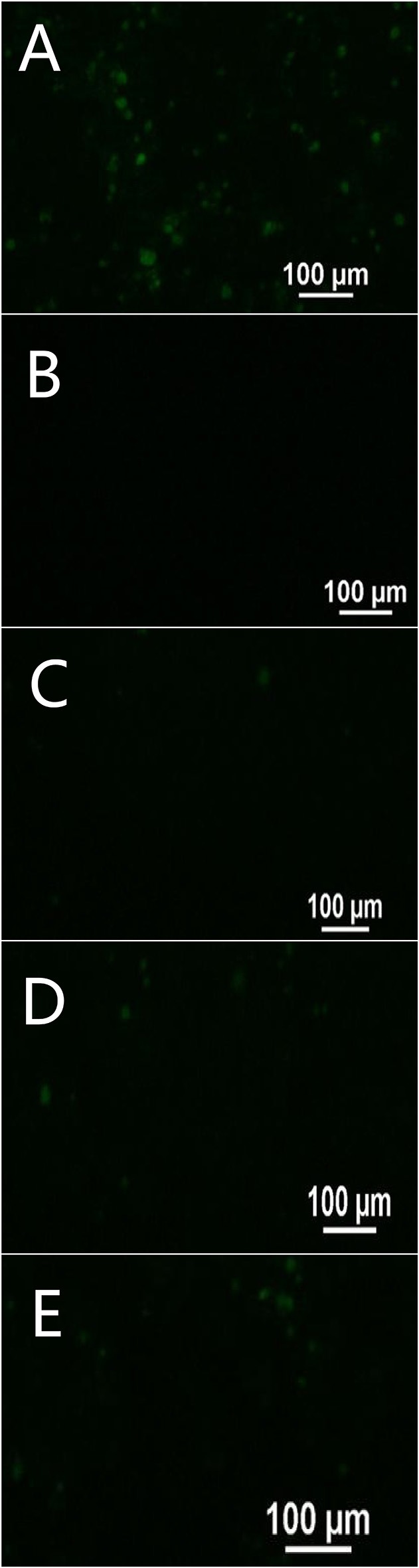
Transport of FITC- PoIFN-α across Caco-2 cell monolayer at 30 min **(C)**, 60 min **(D)**, and 90 min **(E)**. Positive control **(A)** showing FITC- PoIFN-α across the transwell porous membrane without Caco-2 cell monolayers and negative control **(B)** showing HBSS across the Caco-2 cell monolayers on the transwell porous membrane. Green fluorescence spots represents Dye loaded PoIFN-α.

To investigate the absorption mechanism of FITC-PoIFN-α across Caco-2 cell monolayer, the FITC-PoIFN-α was applied when the TEER values reached 300 Ω.cm^2^ (Wang et al., 2008). Then, the transcellular delivery of FITC- PoIFN-α was measured. In Figure 9A, there were strong FITC signals in the basolateral compartment suggesting that the transwell porous membrane could not hamper the permeation of FITC-PoIFN-α. In contrast, there was no FITC signal in Figure 9B, and it excluded non-specified signals. Also, the weak FITC signals were observed till 90 min in Figures 9C–E, indicating that the transportion of FITC-PoIFN-α across the Caco-2 cell monolayer was limited. As reported previously, protein and peptide drugs of large molecular size would be disadvantageous for the absorption (Oda et al., 2011). The molecule weight of PoIFN-α is about 2.5 KD belonging to the protein drug category. Therefore, the low efficiency of transport for PoIFN-α is reasonable. On the other hand, the paracellular pathway is composed of aqueous channels where hydrophilic molecules may diffuse through tight junctions. The tight junctions are composed of transmembrane and cytosolic protein, and they restrict the diffusion of large molecules with a molecular size cutoff around 0.3–1.1 nm (Sadeghi et al., 2008). The radius of PoIFN-α monomer (molecular weight 25 KD) is above 2 nm, which may lead to a limited paracellular transport of IFN-α *in vivo*. Lastly, the tight junction of Caco-2 cell monolayer model is higher than intestinal epithelium *in vivo* (Beduneau et al., 2014). Namely, the intracorporal efficiency of PoIFN-α transport may improve.

## Conclusion

In conclusion, the absorption of FITC- PoIFN-α in the Caco-2 cells was likely time and concentration dependence, and partially energy dependence. The lipid/raft caveolae-mediated pathway might be the most likely endocytic pathway of FITC-PoIFN-α. Then, Golgi complex and lysosomal were involed in localization of FITC-PoIFN-α in Caco-2 cells. Additionally, exocytosis of FITC-PoIFN-α was significantly regulated by COX. Finally, the transcytosis of FITC-PoIFN-α in Caco-2 cells was low. These results provide important insights for the development of the nanoparticles of PoIFN-α. It can enhance the permeability in biomembrane and then improve the bioavailability of loaded PoIFN-α, showing the huge potential for the application in oral pharmaceutical preparations.

## Author contributions

LY designed the whole experiment; XL and SZ directed the completion of the experiment; YQ, WD, YT, XC, YW, and XC provided help during the experiment.

### Conflict of interest statement

The authors declare that the research was conducted in the absence of any commercial or financial relationships that could be construed as a potential conflict of interest.
