# Supplementary material for: Experimental Evaluation of the Transport Mechanisms of PoIFN-α in Caco-2 Cells
Source: Front Pharmacol. 2017 Nov 7;8:781. doi: 10.3389/fphar.2017.00781 (PMC5681924; doi:10.3389/fphar.2017.00781)
Supplement: Supplementary file 1 [file Table1.docx]

Supplement Files

| 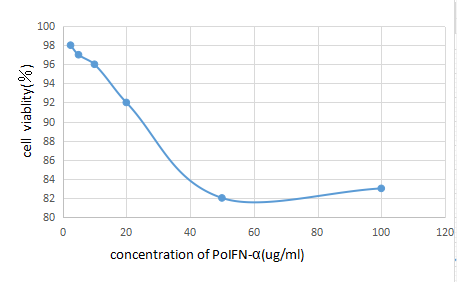  Figure 1 Influence of different concentrations (0,20,40,60,80,100 µg/mL) of FITC-PoIFN-α on Caco-2 cells viability. |
| --- |

**Table 1**

The ratio of activity of alkaline phosphatase in the both side of Caco-2 cell on the transwell during the 21days.

| Time(D) AL/BL(activity of alkaline phosphatase) |
| --- |

7 1.5

14 3.05

21 3.1


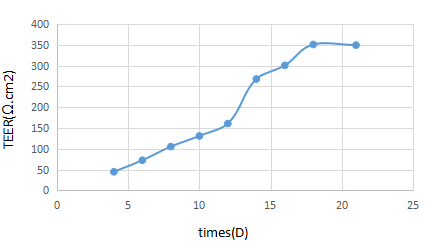


Figure 2 Changes in transepithelia electrical resistance (TEER) values with time in Caco-2 cells during 21 days.
